# High-Confidence Placement of Fragments into Electron Density Using Anomalous Diffraction—A Case Study Using Hits Targeting SARS-CoV-2 Non-Structural Protein 1

**DOI:** 10.3390/ijms241311197

**Published:** 2023-07-07

**Authors:** Shumeng Ma, Vitaliy Mykhaylyk, Matthew W. Bowler, Nikos Pinotsis, Frank Kozielski

**Affiliations:** 1 School of Pharmacy, University College London, 29–39 Brunswick Square, London WC1N 1AX, UK; shumeng.ma.20@ucl.ac.uk; 2 Diamond Light Source, Harwell Science and Innovation Campus, Chilton, Didcot OX11 0DE, UK; vitaliy.mykhaylyk@diamond.ac.uk; 3 European Molecular Biology Laboratory, 38000 Grenoble, France; mbowler@embl.fr; 4 Institute of Structural and Molecular Biology, Birkbeck College, London WC1E 7HX, UK; n.pinotsis@mail.cryst.bbk.ac.uk

**Keywords:** SARS-CoV-2, COVID-19, non-structural proteins, nsp1, tunable wavelength, fragment orientation, anomalous difference Fourier map

## Abstract

The identification of multiple simultaneous orientations of small molecule inhibitors binding to a protein target is a common challenge. It has recently been reported that the conformational heterogeneity of ligands is widely underreported in the Protein Data Bank, which is likely to impede optimal exploitation to improve affinity of these ligands. Significantly less is even known about multiple binding orientations for fragments (<300 Da), although this information would be essential for subsequent fragment optimisation using growing, linking or merging and rational structure-based design. Here, we use recently reported fragment hits for the SARS-CoV-2 non-structural protein 1 (nsp1) N-terminal domain to propose a general procedure for unambiguously identifying binding orientations of 2-dimensional fragments containing either sulphur or chloro substituents within the wavelength range of most tunable beamlines. By measuring datasets at two energies, using a tunable beamline operating in vacuum and optimised for data collection at very low X-ray energies, we show that the anomalous signal can be used to identify multiple orientations in small fragments containing sulphur and/or chloro substituents or to verify recently reported conformations. Although in this specific case we identified the positions of sulphur and chlorine in fragments bound to their protein target, we are confident that this work can be further expanded to additional atoms or ions which often occur in fragments. Finally, our improvements in the understanding of binding orientations will also serve to improve the rational optimisation of SARS-CoV-2 nsp1 fragment hits

## 1. Introduction

Although various biophysical methods exist, X-ray crystallography is considered the gold standard in fragment-based drug discovery (FBDD) as it can instantaneously provide detailed molecular information about the exact binding configuration of fragments [1,2]. This can then be exploited for subsequent rational structure-based drug design [3]. However, fragments are small (<300 Da) and due to their size, it can be challenging to fit these into experimental electron density, a task that can be particularly aggravated at low resolution. However, even at high resolution (<2.0 Å), fragments can still be ambiguous to fit, in particular when they are mostly planar and substituents have not yet been optimised for stronger binding and defined interactions with residues in the binding site. Electron density from fragment binding to proteins can also be incomplete, or weak due to low occupancy of the fragment hit, further complicating interpretation of the experimental map. In the best cases there may only exist a single binding conformation but several orientations may be present at the same time [4,5]. However, before engaging in structure-based rational design of fragment hits, it is essential to possess precise and accurate knowledge about the exact binding conformation to apply rational drug design techniques.

Recently, we reported our first fragment hits targeting SARS-CoV-2 non-structural protein 1 (nsp1) using fragment-based screening via X-ray crystallography [6,7]. Nsp1 is a two-domain protein with a short C-terminal domain, which is involved in the shutdown of host expression by blocking the entry of host mRNA in the ribosome [8]. The C-terminal domain is connected via a linker region with the larger N-terminal domain, which is thought to be involved in circumventing the shutdown of expression of viral mRNA [9] and stabilising the binding of the C-terminus to the ribosome [10]. Therefore, nsp1 is considered as a potential target for antiviral drug discovery.

**Figure 1:**
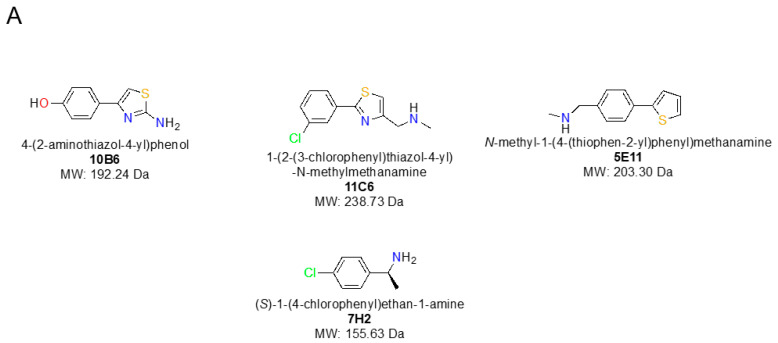

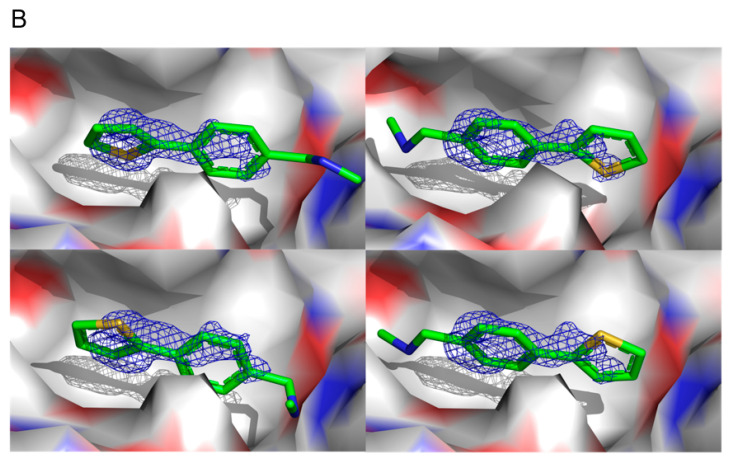
(**A**) Chemical structures of the four nsp1-targeting fragment hits containing sulphur atoms and chloro substituents. (**B**) Schematic representation of the four distinct orientations planar fragments can adopt in suboptimal real-life 2mF_o_-DF_c_ maps (rmsd = 1.0, blue mesh), exemplified by 5E11. One hundred and eighty-degree rotations from one orientation to another can occur along horizontal or vertical axes, in particular when substituents are in quasi symmetric positions on one of the ring systems or if these possess some flexibility and are therefore not visible in the electron density.

All fragment hits were essentially planar (Figure 1A) with non-optimised substituents allowing, in principle, up to four distinct binding orientations in the electron density (Figure 1B). Although working at resolutions between 1.1 and 1.4 Å, calculation of R_free_ values, taking into account atomic B-factors of fragments and careful inspection of electron density for all four configurations did not lead to an absolutely confident identification of a single correct orientation of the fragment hits. Therefore, the most reasonable fragment conformations have been reported [6,7].

To close this knowledge gap with respect to our project on SARS-CoV-2 nsp1, but also to establish a general procedure for similar cases, we used the long-wavelength beamline I23 at Diamond Light Source (DLS). Our aim was to clarify the exact orientation of four out of the six nsp1 fragment hits, containing sulphur and chlorine atoms by measuring diffraction data at wavelengths of 4.509 Å and 2.755 Å. The data collection at lower energy, just below the absorption edge of chlorine, enables the detection of the exact location of anomalous scatterers i.e., sulphur and chlorine, both present in our fragment hits, to be determined. I23 is a tunable beamline where the X-ray diffraction experiment is optimised for data collection at very low energies (long wavelengths). At this beamline, the measurements are carried out in a vacuum environment using a multi-axis goniometer equipped with a cylindrically shaped detector [11].

Using sulphur and chlorine anomalous difference Fourier maps, we report the unambiguous identification of the binding orientation of four nsp1-targeting fragment hits. We show that one fragment is able to simultaneously bind in two distinct orientations, albeit at distinct occupancies. Based on our results we propose a general procedure for unambiguously identifying binding orientations of 2-dimensional fragments containing either sulphur or chloro substituents as starting points for further optimisation for fragments, with substituents that can be examined within the wavelength range of most tunable beamlines.

## 2. Results

Data collection and refinement statistics are summarised in Table 1 and Table 2. The very high I/σ*I* in the highest resolution shell (3.0 to 19.7) can in part be explained by the high resolution of the crystals but also with the low background during data collection. This is due to the complete absence of X-ray scattering in vacuum, which is typically a major cause of the background noise recorded at other macromolecular crystallography beamlines. First, we measured X-ray fluorescence spectra of the sample to identify chemical ions of interest that might be in the crystal or potentially be bound to the protein. The emission spectrum measured at excitation with 4.5 keV exemplified by the nsp1-11C6 complex shows clear peaks assigned to K_a_ lines of sulphur and chlorine at 2.308 and 2.622 keV, respectively (Figure S1).

**Figure 2:**
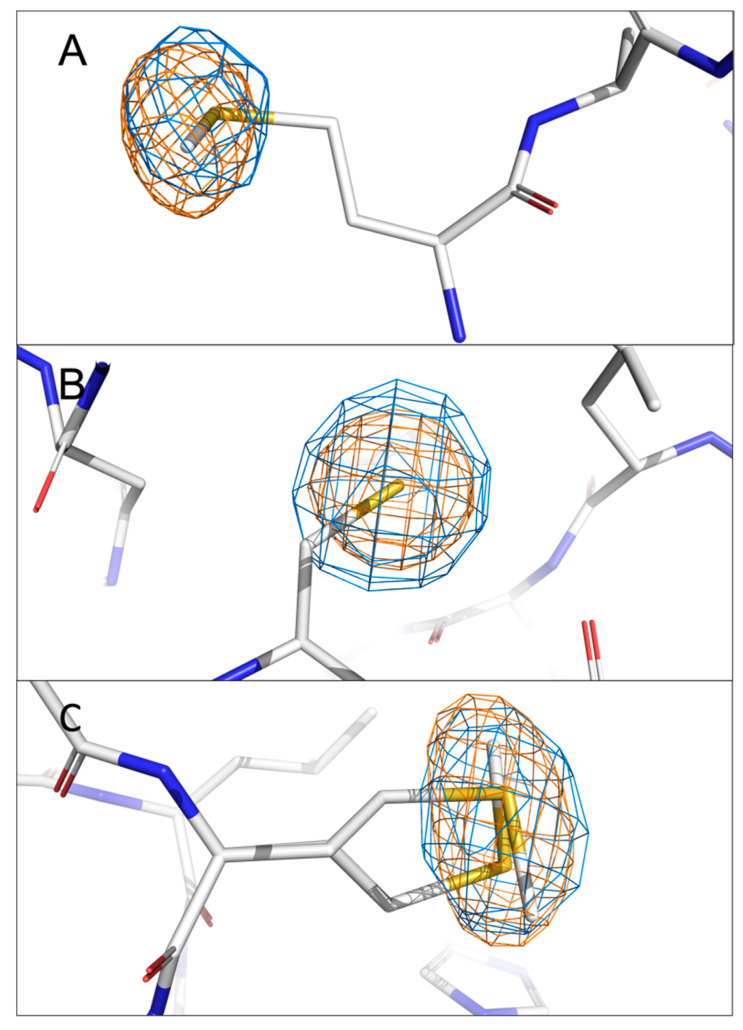
Validation of the localisation of anomalous signals of sulphur from (**A**) Met9, (**B**) Cys51 and (**C**) Met85 in the anomalous difference Fourier maps (rmsd = 4.0) of the nsp1-7H2 complex. The anomalous peak at 4.50 keV is coloured in orange, while that at 2.75 keV is shown in marine. Sulphur, nitrogen, and oxygen atoms in amino acids are coloured in yellow, blue and red, respectively for Figure 2, Figure 3, Figure 4, Figure 5 and Figure 6. The two alternative conformations of Met85 are displayed and correlated well with the slightly elongated form of the anomalous signals.

To validate our system of locating the chlorine and sulphur atoms in anomalous difference Fourier maps at two distinct energies, the anomalous difference Fourier maps of sulphur from methionine and cysteine side chains present in nsp1 were visually inspected. There are two methionines and one cysteine in the N-terminal domain of nsp1. The anomalous signals originating from the sulphur atoms in these residues are visible in both 4.5 keV and 2.75 keV anomalous difference Fourier maps except for nsp1-11C6, for which the anomalous signal of sulphur in Met9 was not detected at 2.75 keV. This is possibly because of the low completeness of the specific data set and the flexibility of Met9 as the first residue at the N-terminus. The signal levels of the sulphur atoms in these residues for all four datasets are listed in Table 3. The overlapping local electron density maps for these residues are exemplified using the nsp1-7H2 complex at two diffraction energies (Figure 2). Interestingly, the first amino acid Met9 was previously placed in a somewhat uncertain position due to its side chain flexibility and therefore lack of electron density for certain side chain atoms. However, the anomalous signal of the sulphur atom clearly reveals its correct localisation (Figure 2A). There is only a single position for the Cys51 side chain, supported by the spherical electron density of the anomalous signal (Figure 2B). The two alternative conformations of the Met85 side chain built in the previous complex (PDB entry 8AYW) structure are also clearly supported by the elongated anomalous sulphur signal (Figure 2C). These results not only validate the reliability of our approach but also show the significance of anomalous difference Fourier maps in revealing missing details in native (non-anomalous) electron-density maps.

**Figure 3:**
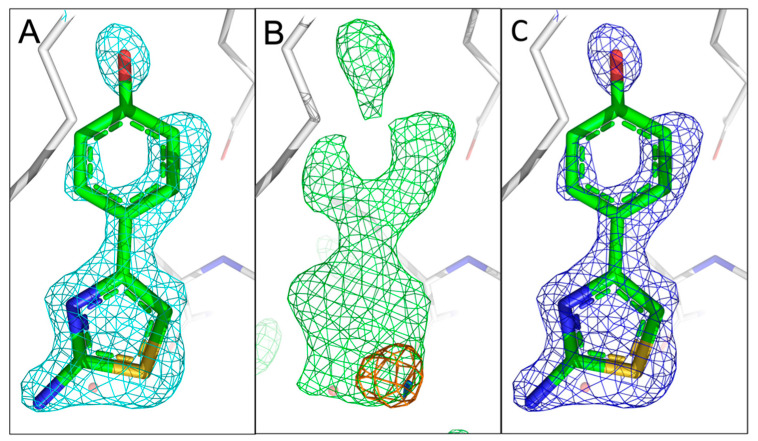
Comparison of the fragment binding site of the previously reported conformation of 10B6 with that obtained using sulphur anomalous difference Fourier maps. (**A**) The published configuration of 10B6. The 2mF_o_-DF_c_ map of the fragment is shown in cyan. (**B**) Anomalous difference Fourier maps calculated from data collected at 4.5 keV (orange) and 2.75 keV (marine) with the mF_o_-DF_c_ map (green) in the fragment region. (**C**) Refined 2mF_o_-DF_c_ map of 10B6 with sulphur atom sitting in the centre of the anomalous peak. The rms deviations for the 2mF_o_-DF_c_, mF_o_-DF_c_ and anomalous difference Fourier maps are 1.0, 3.0, and 4.0, respectively, for Figure 3, Figure 4, Figure 5 and Figure 6. The electron density mostly accounts for 10B6.

Fragment 10B6 (Figure 1A) consists of two rings, containing a heterocyclic 5- and an aromatic 6-ring system. It contains a single sulphur atom in the 2-aminothiazol ring. Only a single anomalous peak is visible at the binding pocket in the 4.5 keV anomalous difference Fourier map at a contour level of 4.0 rmsd. Having inspected the measured anomalous difference Fourier map, we concluded that a value of >4.0 can be adopted as a positive indication of the presence of the atom. The signal is still visible in the 2.75 keV anomalous difference Fourier map (rmsd of 4.0), albeit a smaller peak, confirming the existence of sulphur in fragment 10B6, corresponding to its chemical structure. We could easily fit 10B6 into the density with the sulphur atom sitting in the centre of the sulphur peak. A comparison with our recently published fragment configuration shows that our previous assumption was correct, and we only observed a single configuration in the difference map (Figure 3A–C).

**Figure 4:**
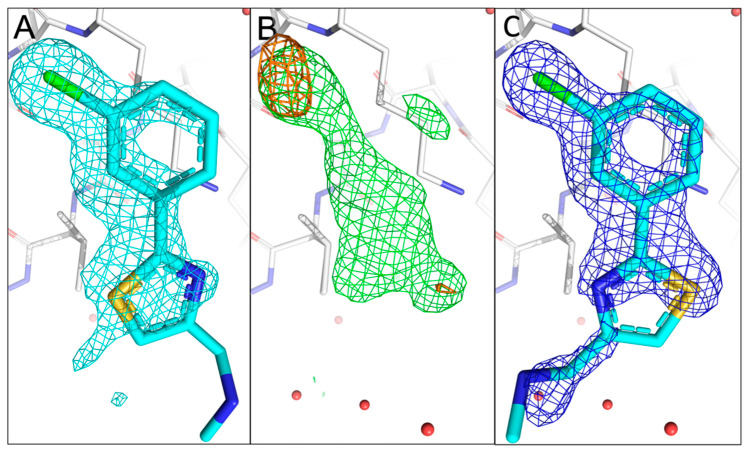
Comparison of the fragment binding site of the previously reported conformation of 11C6 with that obtained using sulphur and chlorine anomalous difference Fourier maps. (**A**) The published configuration of 11C6. The 2mF_o_-DF_c_ map of the fragment is shown in cyan. (**B**) Anomalous difference Fourier map calculated from data collected at 4.5 keV (orange) with the mF_o_-DF_c_ map (green) in the fragment binding region. The anomalous peak from 2.75 keV data does not appear in the sulphur location possibly because of the low occupancy of 11C6. (**C**) Refined 2mF_o_-DF_c_ map of 11C6 with the chlorine and sulphur atoms sitting in the centres of the anomalous peaks. The electron density only partially covers 11C6, but the orientation of the fragment hit could be confirmed by the anomalous signals for sulphur and chlorine.

Fragment 11C6 can be considered as a closely related analogue of 10B6 (Figure 1A). The sulphur atom is located in a thiazole ring system, and it also contains a chloro substituent in the *meta*-position of the phenyl group, allowing us to observe two anomalous signals in the experimental anomalous difference Fourier map. Indeed, two anomalous peaks are visible in the fragment binding site in the 4.5 keV anomalous difference Fourier map (rmsd of 4.0). Neither of the signals is visible in the 2.75 keV anomalous difference Fourier map even at contour level of 3.0 rmsd, which is expected for the chlorine- but not for the sulphur atom in 11C6. This could be caused by the lower occupancy of 11C6 in the binding site or the overall weak anomalous signals at lower energy maps as observed for sulphur anomalous signals from Met9. However, as one anomalous peak is located approximately at an extension of the ligand density while the second one is slightly away from the main ligand density, we can easily distinguish and allocate the sulphur-containing ring as well as the chloro substituent, making unambiguous fragment orientation and conformation possible (Figure 4A–C).

**Figure 5:**
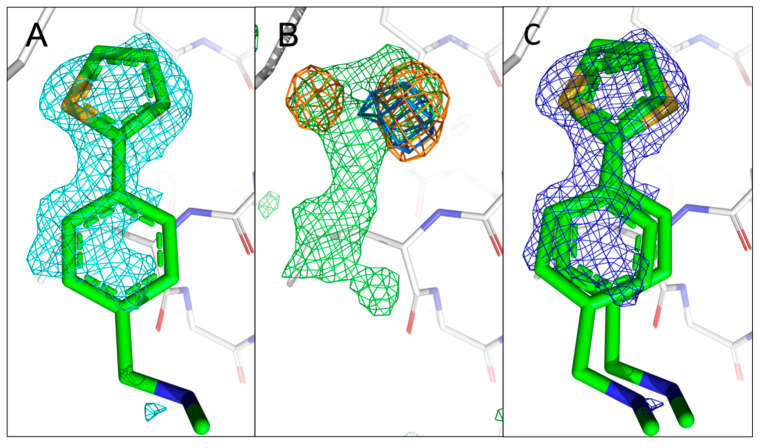
Comparison of the previously reported single conformation of 5E11 with those obtained using sulphur anomalous difference Fourier maps. (**A**) The published configuration of 5E11. The 2mF_o_-DF_c_ map of the fragment is shown in cyan. (**B**) Anomalous difference Fourier maps calculated from datasets collected at 4.5 keV (orange) and 2.75 keV (marine) with the mF_o_-DF_c_ map (green) in the fragment region. (**C**) Refined 2mF_o_-DF_c_ map of 5E11 in the two orientations with sulphur sitting in the centres of the two anomalous peaks obtained from 4.5 keV data. The electron density mostly covers 5E11 in both distinct orientations, but there is no density around the N-methyl-methanamine substituent, indicating its flexibility and complicating unambiguous placement of the fragment in the absence of sulphur peaks.

Compared to the previous two fragments hits, 5E11 (Figure 1A) represents a much smaller fragment structure containing a single N-methyl-methanamine substituent in the *para*-position of the phenyl ring system with the sulphur atom being part of a thiophen ring system. To our surprise, for 5E11, two anomalous peaks are visible at the fragment binding site in the 4.5 keV anomalous difference Fourier map (rmsd of 4.0), albeit with different intensities. The two signals are still visible in the 2.75 keV anomalous difference Fourier map (rmsd of 3.0), confirming the existence of sulphur in 5E11 (Figure S2), in agreement with its chemical structure. However, this also implies two simultaneous binding conformations with distinct occupancies. In our previous work, we had fitted 5E11 in a single orientation as the electron density even at very high resolution did not reveal multiple orientations. However, our new more detailed approach employed here clearly reveals two orientations for the sulphur atom. Therefore, we manually fitted 5E11 in two distinct orientations with different occupancy (Figure 5C). This is an excellent example of how to use anomalous difference data to identify all possible binding orientations of a fragment before engaging in SAR-by-catalogue or by synthesising novel fragment analogues.

**Figure 6:**
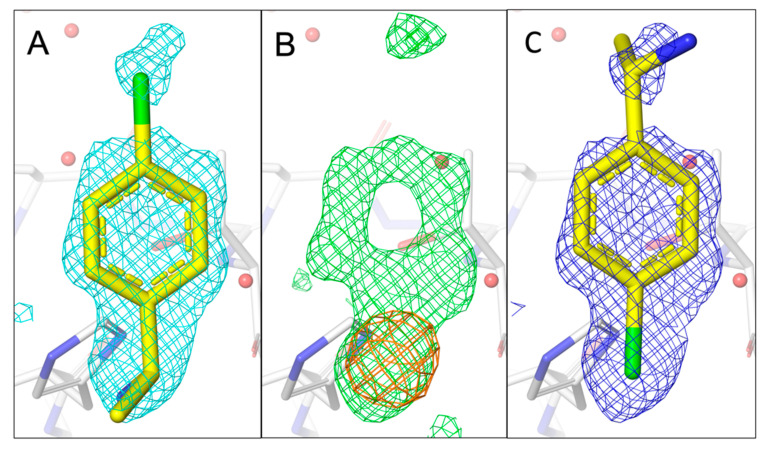
Comparison of the fragment binding site of previously reported conformation of 7H2 with that obtained using a chlorine anomalous difference Fourier map. (**A**) The published configuration of 7H2 with the 2mF_o_-DF_c_ map around the fragment is shown in cyan. (**B**) Anomalous difference Fourier map calculated from data collected at 4.5 keV (orange) together with the mF_o_-DF_c_ map (green) in the fragment binding region. (**C**) Refined 2mF_o_-DF_c_ map of 7H2 with the chlorine located in the centre of the anomalous peak. The electron density mostly covers 7H2.

Whereas the previous three fragment hits can be considered as chemically closely related analogues, 7H2 represents a smaller fragment of 155 Da containing a single central phenyl ring with two distinct substituents binding to a shallower site on nsp1 (Figure 6A–C). Due to its quasi-symmetrical structure (Figure 1A), a rotation around its long axis does not lead to a new distinct orientation, as the ethan-1-amine substituent contains a rotatable bond. However, a vertical axis through its phenyl ring will lead to two distinct orientations with each of the two substituents changing positions. For 7H2, only one anomalous peak at the binding pocket is visible in the 4.5 keV anomalous difference Fourier map at rmsd of 4.0. The peak disappears in the 2.75 keV anomalous difference Fourier map (same rmsd level), consistent with the existence of a single chlorine in 7H2. Consequently, we fitted 7H2 into the density with the chloro substituent located at the centre of the anomalous peak, in the opposite direction of our previously published orientation.

## 3. Discussion

The unambiguous identification of atomic elements from phased anomalous difference Fourier maps relies on the measurements above and below an absorption edge of the atom. This technique is shown to be very useful in unravelling the structure and function of ion-binding proteins, but it has been challenging for the lighter atomic weight elements at standard synchrotron beamlines due to air absorption as well as other technical constraints [12]. The DLS I23 beamline operates in vacuum and is optimised for the data collection at wavelengths up to 5.9 Å enabling it to reach K-absorption edges of P, S, Cl, K, and Ca (Figure S2). The absence of scattering from the air results in a very low endogenous background of the detector that translates into the improved signal-to-noise ratio in the anomalous difference Fourier maps. Furthermore, due to a much-enhanced value of f’’ at L- absorption edges of other biologically relevant halides, i.e., Br and I substituents, the identification of their position is more straightforward when measured at I23 [13]. Consequently, by conducting experiments around the absorption edges of interest located in this energy range one can determine the identity and location of relevant substituents in fragments that bind to proteins and then use this information for fitting in the electron density map. Therefore, undertaking these long wavelength experiments is especially useful and recommended when there is an ambiguous placement into challenging electron density.

A previous study revealed that less than 2% of entries containing small molecules/ligands reported two or more alternate conformations [1], which is probably a consequence of past limitations such as beamline design, a vacuum environment to reduce noise and dedicated detectors. The available published information on ligand orientation or conformation is even rarer for fragment hits with molecular weights of <300 Da, corresponding to less than 20 non-hydrogen atoms. The extent of simultaneously existing multiple orientations of fragments binding to the same site may be even more frequent as their molecular weight is significantly smaller than that of small molecules with suboptimal interactions with residues in the binding pocket at the start of a campaign. Planar fragments that lack 3D characteristics, or those with low occupancy and/or flexible sub-optimised ligands can now be confidently placed. The “flatness” of fragments has been recognised as a challenge and various attempts have been made to incorporate fragments with 3D characteristics into libraries [14,15]. Therefore, it is not surprising that studies involving anomalous difference maps for fragments are rare compared to small molecule work. Brominated fragments have been preferentially employed, likely due to their easy use as they can be measured using even X-ray generators and do not require tunable beamlines. For example, in a pilot study, brominated fragments have been employed to identify fragment hits at low resolution [16]. A library with 68 brominated fragments was screened against HIV protease to successfully identify binding sites and fragment orientations [17]. In another project the authors employed brominated and chlorinated fragment analogues using a tunable beamline to locate novel fragment binding sites in glycerol-3-phosphate dehydrogenase and to identify two distinct binding orientations [18]. In our work, we provide an optimised protocol using a dedicated tunable beamline to focus on sulphur and chlorine containing substituents in fragment hits.

The long-wavelength MX beamline I23 operating in a vacuum environment has made this type of study possible, allowing for experimentation in proximity to the absorption edges of sulphur and chlorine. Additionally, this controlled setting effectively reduces noise levels, optimizing the detection of anomalous difference signals.

A recent study showed that 24% of marketed drugs contained sulphur atoms and approximately 28% were halogenated [19]. Halogen-enriched fragment libraries with fragments containing at least one halogen atom per fragment are available [20], which have been created because of the potential to form halogen-bonds with nucleophilic atoms or groups in proteins. We probed recently identified fragment hits obtained by fragment-based screening via X-ray crystallography [6]. The resulting fragment hits were predominantly planar due to aromatic ring systems and despite working at resolutions between 1.1 and 1.4 Å, electron density maps remained ambiguous, even after extensive refinement. Not unexpectedly, neither R_free_ values, density maps or atomic B-factors were fully conclusive raising our suspicion of the presence of multiple fragment orientations. Intuition led to the correct placement of two out of four fragment hits, but intuition should not be the basis for subsequent rational structure-based design. Consequently, we identified fragment 5E11 that simultaneously binds to nsp1 in two distinct orientations as revealed by two peaks in the anomalous difference Fourier maps. Based on their intensity we could also estimate the occupancy for each orientation with a preference of the sulphur atom in the thiophene ring pointing towards the protein surface and establishing hydrogen bond interaction with Lys47 [6] rather than pointing towards the solvent. As both sulphur difference peaks are located on the same ring, our approach is powerful enough to pinpoint individual atoms on a five-membered ring system with a distance of only 2.6 Å between them. A further improvement of our approach could be to measure a third dataset at a shorter wavelength to further improve data resolution.

Based on our work we recommend the following procedure for data collection of planar fragments with ambiguous or weak electron density due to low occupancy or the presence of multiple orientations. First collect data at the highest possible resolution. If doubts exist about the correct orientation or conformation of the fitted fragment making rational fragment optimisation a challenge, use fragment analogues with detectable substituents such as sulphur and chlorine by measuring anomalous difference data above and below the absorption edge of the atom at a tunable beamline to clearly locate it orientation. If radiation damage is not an issue, data collection can include a native dataset at high resolution measured on the same crystal. Although currently limited to sulphur and chlorine-containing fragments, work is currently in progress to expand our approach to other elements frequently present in fragments.

## 4. Materials and Methods

### 4.1. Expression, Purification and Crystallisation of SARS-CoV-2 nsp1_10-126_

The N-terminal domain of SARS-CoV-2 nsp1_10-126_, containing residues 10 to 126 and named nsp1 throughout the manuscript, was expressed, purified and crystallised as previously described [6]. The crystallisation condition used is Index 71 (Cat. No.: HR2-944-71) 0.1 M BIS-TRIS pH 6.5, 0.2 M NaCl and 25% *w*/*v* Polyethylene glycol 3350 from Hampton Research, Aliso Viejo, CA, USA.

Fragment hits 5E11, 10B6, and 11C6 were obtained from the Maybridge Ro3 library, while 7H2 was purchased from Molport, Beacon, NY, USA (Cat. No.: HY-30379). Each of them was dissolved in DMSO-d6 (Sigma, St. Louis, MO, USA; CAS Number: 2206-27-1) at a concentration of 200 mM as a stock solution. An amount of 2 μL of each stock solution was mixed with 8 μL of the final buffer (10 mM HEPES pH 7.6 and 300 mM NaCl) (Sigma, St. Louis, MO, USA; CAS Number: 7365-45-9 and 7647-14-5), giving a final concentration of 40 mM fragment containing 20% DMSO-d6. Each fragment solution (1.5 μL) was added into approximately 1 μL of crystallisation drops, making a final fragment concentration of 24 mM and approximately 12% DMSO-d6. These drops were incubated at room temperature for 4–5 h followed by crystal harvesting using loops, cryo-cooled in liquid nitrogen and stored in pucks for sample storage and shipment.

### 4.2. Data Collection, Structure Determination and Refinement

The long-wavelength diffraction experiments were carried out at DLS, beamline I23. Each sample was subjected to X-rays at energies of 4.50 keV and 2.75 keV (corresponding to wavelengths of 2.755 Å and 4.509 Å), above and below the chlorine K absorption edge of 2.82 keV but both being above the sulphur K absorption edge of 2.47 keV. Diffraction experiments were performed in vacuum using the semi-cylindrical PILATUS 12M detector. During the measurements the temperature of protein crystals mounted on copper sample holders was estimated to be at ca. 80 K.

For each sample a 360-degree rotation and fine-slicing (0.10°) dataset was collected to obtain high multiplicity with the resolution only limited by detector dimensions (1.8 Å and 2.9 Å at wavelength 2.755 Å and 4.509 Å, respectively).

The data processing pipelines fast_DP [v1.6.2] and Xia2 [v3.12.0], including Xia2_3dii and Xia2_dials [21], were automatically employed. PDB entry 8A55 and the protein sequence were provided in ISPyB to trigger the processing pipeline Dimple [v2.6.2] to generate anomalous difference Fourier maps and MrBUMP [v2.2.6] to find a molecular replacement solution. Within the Dimple run, ten cycles of rigid-body refinement were performed, followed by four cycles of jelly body refinement and eight cycles of restrained refinement before starting to identify anomalous difference peaks [22].

To identify unambiguous binding orientations of the four fragment hits, the nsp1-fragment coordinates and anomalous difference Fourier maps were overlaid with four nsp1 2mF_o_-DF_c_ and mF_o_-DF_c_ maps, as previously reported [6], and inspected in COOT [v8.0] [23]. For 5E11, 10B6 and 11C6, the binding site is located in proximity to residue Lys125, whereas for 7H2, the shallower binding site is located adjacent to Pro109, as recently described [6]. Fragment hits were manually refitted into the ligand density with sulphur and (or) chlorine sitting in the centres of their anomalous difference Fourier map peaks. The updated structures were iteratively refined in Phenix [v1.20.1-4487] [24] and inspected in COOT. Figure 1A was prepared using Chemdraw [v20.1] [25], whereas Figure 1B, Figure 3, Figure 4, Figure 5 and Figure 6 were generated in Pymol [v2.4.1] [26], and Figure 1B shows screenshots captured in COOT.

## Figures and Tables

**Table 1:** Data collection statistics for four nsp1-fragment hit complexes measured at two distinct wavelengths. Data in the highest shell are shown in parenthesis. * These previous PDB entries have been replaced in Table 2 with new ones containing the updated coordinates.

	Nsp1-5E11	Nsp1-7H2	Nsp1-10B6	Nsp1-11C6
Previous PDB Entries	8ASQ *	8AYW *	8AYS	8AZ9 *
Wavelengths [Å]	2.755/4.509			
Resolution range [Å]	141.36–1.80 (1.83–1.80)/ 35.61–2.90 (2.95–2.90)	35.63–1.77 (1.80–1.77)/ 36.83–2.90 (2.95–2.90)	141.16–1.80 (1.83–1.80)/ 36.82–2.90 (2.95–2.90)/	36.65–1.77 (1.80–1.77)/ 35.47–2.90 (2.95–2.90)
Space group	P4_3_2_1_2			
Unit cell parameters [Å, °]	*a* = *b* = 36.8, *c* = 141.4, α = β = γ = 90	*a* = *b* = 36.8, *c* = 142.2, α = β = γ = 90	*a* = *b* = 36.8, *c* = 141.2, α = β = γ = 90	*a* = *b* = 36.7, *c* = 140.4, α = β = γ = 90
Total reflections	136,290 (2902)/ 24,887 (377)	112,793 (1811)/ 24,966 (378)	135,156 (2719)/ 25,288 (390)/	111,277 (1602)/ 24,459 (390)
Unique reflections	9806 (480)/ 2501 (103)	8900 (320)/ 2225 (70)/	9742 (467)/ 2470 (100)/	7149 (237)/ 1753 (57)
Multiplicity	13.9 (6.0)/ 10.0 (3.7)	12.7 (5.7)/ 11.2 (5.4)/	13.9 (5.8)/ 10.2 (3.9)/	15.6 (6.8)/ 14.0 (6.8)
Completeness [%]	100.0 (99.4)/ 100.0 (95.4)	87.0 (63.6)/ 89.1 (63.1)/	100.0 (98.3)/ 99.6 (90.1)/	71.3 (47.8)/ 71.8 (49.1)
Anomalous completeness [%]	99.9 (98.0)/ 98.8 (90.0)	85.3 (62.5)/ 86.3 (60.0)	99.9 (98.0)/ 99.0 (91.9)	72.5 (48.6)/ 74.4 (53.3)
Mean I/sigma(I)	63.7 (19.7)/ 25.9 (14.0)	34.3 (7.2)/ 19.3 (8.1)	41.7 (10.7)/ 16.9 (8.9)/	23.2 (3.0)/ 13.8 (5.9)/
Wilson B-factor [Å^2^]	19/19	24/40	17/29	29/44
R-meas [%]	3.3 (10.2)/ 8.3 (8.7)	4.7 (19.4)/ 10.7 (13.3)	4.7 (18.9)/ 12.7 (11.2)/	7.5 (62.6)/ 18.0 (31.4)
CC_1/2_ [%]	100.0 (99.4)/ 99.5 (98.9)	100.0 (98.2)/ 99.2 (99.1)	100.0 (97.3)/ 99.1 (97.5)/	99.3 (75.9)/ 97.1 (92.0)

**Table 2:** Refinement statistics for the three nsp1-fragment complexes. Structure factors used to generate the updated nsp1-fragment complexes were taken from previous data collections. Data in the highest shell are shown in parenthesis.

	Nsp1-5E11	Nsp1-7H2	Nsp1-11C6
PDB ID	8CRF	8CRK	8CRM
R_cryst_/R_free_ [%]	19.2 (30.8)/24.4 (34.6)	16.8 (26.8)/19.2 (27.7)	17.9 (29.2)/20.4 (28.9)
Reflections used in refinement	34,387 (3461)	40,247 (3890)	19,211 (1871)
Reflections used for R_free_	868 (87)	988 (96)	951 (97)
Total no. of non-hydrogen atoms (protein)	1013	1041	1113
No. of protein/ligand/solvent atoms	885/54/100	919/20/112	973/26/125
Average B-factor/protein/ligands/solvent	26.4/23.8/75.8/35.6	22.6/21.1/48.1/32.6	25.3/23.9/54.9/32.9
RMSD (bonds, angles)	0.012/1.30	0.012/1.39	0.012/1.27
Ramachandran favoured/allowed/outliers/rotamer outliers [%]	99.1/0.90/0/1.1	974/2.7/0/1.00	96.6/3.5/0/0.9
Clashscore	5.5	5.3	6.0

**Table 3:** Anomalous signal levels of sulphur from Met9, Cys51 and Met85 in four nsp1-fragment datasets measured at two distinct diffraction energies.

Fragment Hits		5E11	7H2	10B6	11C6
Diffraction Energy (keV)		2.75/4.50			
S (σ)	Met9	8.0/12.7	6.1/11.1	8.0/14.2	n.d./7.4
	Cys51	18.8/46.8	12.7/34.0	14.02/38.6	7.3/21.5
	Met85	10.6/21.6	7.4/17.3	8.5/18.4	5.3/13.1

n.d. not detected.

## Supplementary Materials

The supporting information can be downloaded at: https://www.mdpi.com/article/10.3390/ijms241311197/s1.

## Abbreviations

BIS-TRIS	Bis(2-hydroxyethyl)amino-tris(hydroxymethyl)methane
CoV	Coronavirus
COVID-19	Coronavirus Disease 2019
Da	Dalton
DLS	Diamond Light Source
DMSO	Dimethyl sulfoxide
FBDD	Fragment-based drug discovery
HEPES	(4-(2-hydroxyethyl)-1-piperazineethanesulfonic acid
IPTG	Isopropyl-β-D-thiogalactopyranoside
mRNA	Messenger ribonucleic acid
Nsp	Non-structural protein
PDB	Protein data bank
PMSF	Phenylmethylsulfonyl Fluoride
rmsd	Root Mean Square Deviation
SARS-CoV-2	Severe Acute Respiratory Syndrome Coronavirus

## References

[1] van Zundert G.C.P., Hudson B.M., de Oliveira S.H.P., Keedy D.A., Fonseca R., Heliou A., Suresh P., Borrelli K., Day T., Fraser J.S. (2018). qFit-ligand Reveals Widespread Conformational Heterogeneity of Drug-Like Molecules in X-Ray Electron Density Maps. J. Med. Chem..

[2] Deschamps J.R., Rapaka R.S., Sadée W. (2008). The Role of Crystallography in Drug Design. Drug Addiction: From Basic Research to Therapy.

[3] Davis A.M., Teague S.J., Kleywegt G.J. (2003). Application and Limitations of X-ray Crystallographic Data in Structure-Based Ligand and Drug Design. Angew. Chem. Int. Ed..

[4] Han G.W., Lee J.Y., Song H.K., Chang C., Min K., Moon J., Shin D.H., Kopka M.L., Sawaya M.R., Yuan H.S. (2001). Structural basis of non-specific lipid binding in maize lipid-transfer protein complexes revealed by high-resolution X-ray crystallography1 1Edited by D. Rees. J. Mol. Biol..

[5] Murthy K.H., Winborne E.L., Minnich M.D., Culp J.S., Debouck C. (1992). The crystal structures at 2.2-A resolution of hydroxyethylene-based inhibitors bound to human immunodeficiency virus type 1 protease show that the inhibitors are present in two distinct orientations. J. Biol. Chem..

[6] Ma S., Damfo S., Lou J., Pinotsis N., Bowler M.W., Haider S., Kozielski F. (2022). Two Ligand-Binding Sites on SARS-CoV-2 Non-Structural Protein 1 Revealed by Fragment-Based X-ray Screening. Int. J. Mol. Sci..

[7] Borsatto A., Akkad O., Galdadas I., Ma S., Damfo S., Haider S., Kozielski F., Estarellas C., Gervasio F.L. (2022). Revealing druggable cryptic pockets in the Nsp1 of SARS-CoV-2 and other β-coronaviruses by simulations and crystallography. eLife.

[8] Schubert K., Karousis E.D., Jomaa A., Scaiola A., Echeverria B., Gurzeler L.A., Leibundgut M., Thiel V., Muhlemann O., Ban N. (2020). SARS-CoV-2 Nsp1 binds the ribosomal mRNA channel to inhibit translation. Nat. Struct. Mol. Biol..

[9] Shi M., Wang L., Fontana P., Vora S., Zhang Y., Fu T.-M., Lieberman J., Wu H. (2020). SARS-CoV-2 Nsp1 suppresses host but not viral translation through a bipartite mechanism. Biorxiv Prepr. Serv. Biol..

[10] Mendez A.S., Ly M., Gonzalez-Sanchez A.M., Hartenian E., Ingolia N.T., Cate J.H., Glaunsinger B.A. (2021). The N-terminal domain of SARS-CoV-2 nsp1 plays key roles in suppression of cellular gene expression and preservation of viral gene expression. Cell Rep..

[11] Wagner A., Duman R., Henderson K., Mykhaylyk V. (2016). In-vacuum long-wavelength macromolecular crystallography. Acta Crystallogr. Sect. D.

[12] Handing K.B., Niedzialkowska E., Shabalin I.G., Kuhn M.L., Zheng H., Minor W. (2018). Characterizing metal-binding sites in proteins with X-ray crystallography. Nat. Protoc..

[13] Rocchio S., Duman R., El Omari K., Mykhaylyk V., Orr C., Yan Z., Salmon L., Wagner A., Bardwell J.C.A., Horowitz S. (2019). Identifying dynamic, partially occupied residues using anomalous scattering. Acta Crystallogr. Sect. D.

[14] Morley A.D., Pugliese A., Birchall K., Bower J., Brennan P., Brown N., Chapman T., Drysdale M., Gilbert I.H., Hoelder S. (2013). Fragment-based hit identification: Thinking in 3D. Drug Discov. Today.

[15] Hall R.J., Mortenson P.N., Murray C.W. (2014). Efficient exploration of chemical space by fragment-based screening. Prog. Biophys. Mol. Biol..

[16] Grøftehauge M.K., Therkelsen M.Ø., Taaning R., Skrydstrup T., Morth J.P., Nissen P. (2013). Identifying ligand-binding hot spots in proteins using brominated fragments. Acta Cryst. Sect. F Struct. Biol. Cryst. Commun..

[17] Tiefenbrunn T., Forli S., Happer M., Gonzalez A., Tsai Y., Soltis M., Elder J.H., Olson A.J., Stout C.D. (2014). Crystallographic fragment-based drug discovery: Use of a brominated fragment library targeting HIV protease. Chem. Biol. Drug. Des..

[18] Choe J., Suresh S., Wisedchaisri G., Kennedy K.J., Gelb M.H., Hol W.G. (2002). Anomalous differences of light elements in determining precise binding modes of ligands to glycerol-3-phosphate dehydrogenase. Chem. Biol..

[19] Mirza A., Desai R., Reynisson J. (2009). Known drug space as a metric in exploring the boundaries of drug-like chemical space. Eur. J. Med. Chem..

[20] Heidrich J., Sperl L.E., Boeckler F.M. (2019). Embracing the Diversity of Halogen Bonding Motifs in Fragment-Based Drug Discovery-Construction of a Diversity-Optimized Halogen-Enriched Fragment Library. Front. Chem..

[21] Winter G., Beilsten-Edmands J., Devenish N., Gerstel M., Gildea R.J., McDonagh D., Pascal E., Waterman D.G., Williams B.H., Evans G. (2022). DIALS as a toolkit. Protein Sci..

[22] Wojdyr M., Keegan R., Winter G., Ashton A. (2013). DIMPLE—A pipeline for the rapid generation of difference maps from protein crystals with putatively bound ligands. Acta Crystallogr. Sect. A.

[23] Emsley P., Lohkamp B., Scott W.G., Cowtan K. (2010). Features and development of Coot. Acta Cryst. D Biol. Cryst..

[24] Liebschner D., Afonine P.V., Baker M.L., Bunkoczi G., Chen V.B., Croll T.I., Hintze B., Hung L.-W., Jain S., McCoy A.J. (2019). Macromolecular structure determination using X-rays, neutrons and electrons: Recent developments in Phenix. Acta Crystallogr. Sect. D.

[25] Cousins K.R. (2005). ChemDraw Ultra 9.0. CambridgeSoft, 100. J. Am. Chem. Soc..

[26] Schrodinger L. (2015). The PyMOL Molecular Graphics System, Version 1.8. https://pymol.org/2/.

