# Supplementary material for: High-Confidence Placement of Fragments into Electron Density Using Anomalous Diffraction—A Case Study Using Hits Targeting SARS-CoV-2 Non-Structural Protein 1
Source: Int J Mol Sci. 2023 Jul 7;24(13):11197. doi: 10.3390/ijms241311197 (PMC10342360; doi:10.3390/ijms241311197)
Supplement: Supplementary file 1 [file ijms-24-11197-s001.zip › ijms-2493541-supplementary.pdf]

Supplementary Material Section

High-Confidence Placement of Fragments into Electron Density Using Anomalous Diffraction—A Case Study Using Hits Targeting SARS-CoV-2 Non-Structural Protein 1

Shumeng Ma [1], Vitaliy Mykhaylyk [2], Matthew W. Bowler [3], Nikos Pinotsis [4] and Frank Kozielski [1,*]

[1] School of Pharmacy, University College London, 29–39 Brunswick Square, London WC1N 1AX, UK; shumeng.ma.20@ucl.ac.uk
[2] Diamond Light Source, Harwell Science and Innovation Campus, Chilton, Didcot OX11 0DE, UK; vitaliy.mykhaylyk@diamond.ac.uk
[3] European Molecular Biology Laboratory, 38000 Grenoble, France; mbowler@embl.fr
[4] Institute of Structural and Molecular Biology, Birkbeck College, London WC1E 7HX, UK; n.pinotsis@mail.cryst.bbk.ac.uk
* Correspondence: f.kozielski@ucl.ac.uk

Content

Figure S1 1

Figure S2 2

Figure S1

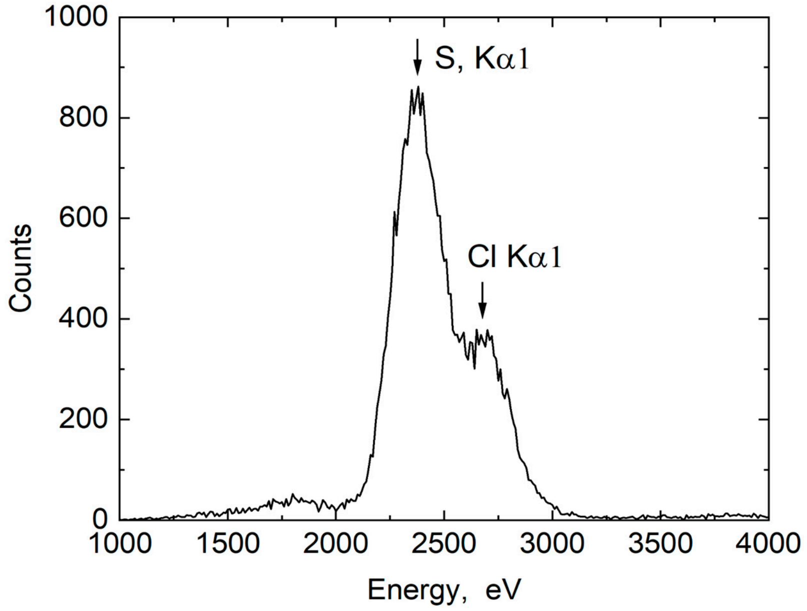

Figure S1. X-ray fluorescence spectra of the nsp1-**11C6** crystal sample. The peaks at 2308 and 2622 keV correspond to K_{a1} emission lines of sulphur and chlorine. The elevation at low energy is a background predominantly caused by X-ray emission of silicon from the detector.

Figure S2

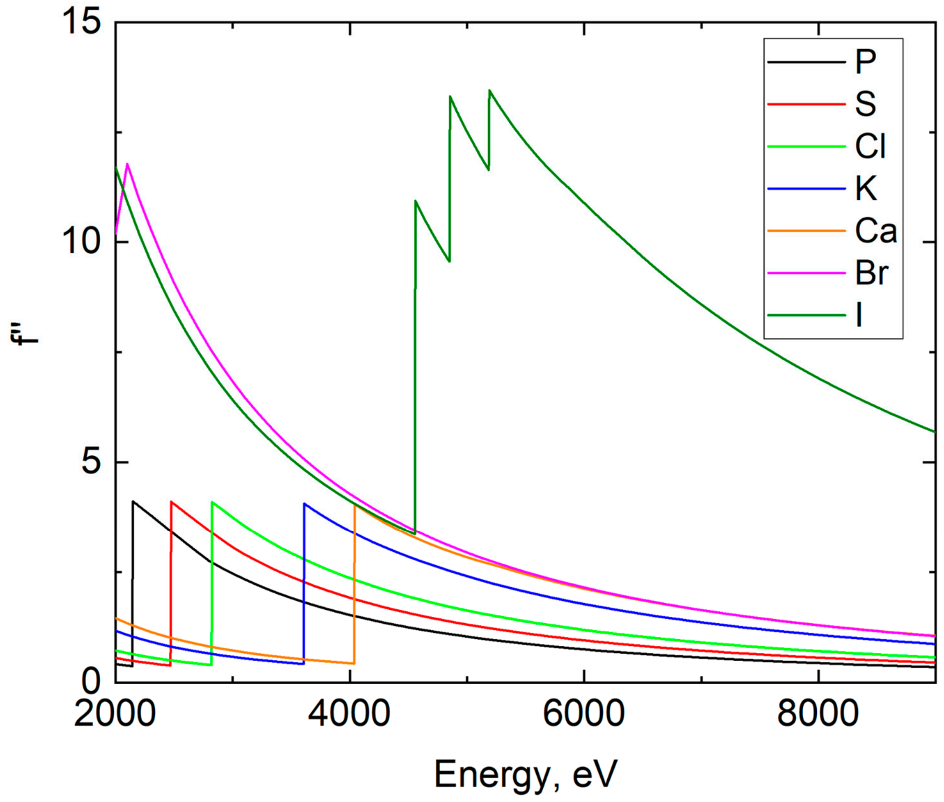

Figure S2. Energy dependence of the anomalous contribution to the structural factor f'' for biologically relevant light ions and halides (data from www.bmsc.washington.edu). The value of f'' manifests a sharp increase at the K-edge of light atoms (P, S, Cl, K, Ca) or L-edges of Br and I thus enabling positive identification of the ions.
